# Supplementary material for: A deletion in the STA1 promoter determines maltotriose and starch utilization in STA1+ Saccharomyces cerevisiae strains
Source: Appl Microbiol Biotechnol. 2019 Jul 26;103(18):7597–615. doi: 10.1007/s00253-019-10021-y (PMC6719335; doi:10.1007/s00253-019-10021-y)
Supplement: Supplementary file 1 — (PDF 1319 kb) [file 253_2019_10021_MOESM1_ESM.pdf]

Supplementary Material

Journal: Applied Microbiology and Biotechnology

Title: A deletion in the *STA1* promoter determines maltotriose and starch utilization in *STA1+* *Saccharomyces cerevisiae* strains

Kristoffer Krogerus[1,2#], Frederico Magalhães[1], Joosu Kuivanen[1*], Brian Gibson[1]

[1] VTT Technical Research Centre of Finland, Tietotie 2, P.O. Box 1000, FI-02044 VTT, Espoo, Finland

[2] Department of Biotechnology and Chemical Technology, Aalto University, School of Chemical Technology, Kemistintie 1, Aalto, P.O. Box 16100, FI-00076 Espoo, Finland

* Current Affiliation: Tampere University, Tampere, Finland

# Address correspondence to Kristoffer Krogerus, kristoffer.krogerus@gmail.com

Supplementary Data

Supplementary Data 1 – The file contains the sequences of the *STA1* open reading frame and upstream region in the 15 *Saccharomyces cerevisiae* strains that were analysed in the study.

Supplementary Data 2 – A81062.ONT_only.final.fa is the *de novo* assembly of *S. cerevisiae* A81062 generated only from reads produced with the MinION (polished with NanoPolish).

Supplementary Data 3 – WY3711.ONT_only.final.fa is the *de novo* assembly of *S. cerevisiae* WY3711 generated only from reads produced with the MinION (polished with NanoPolish).

Supplementary Figures and Tables

Sanger sequencing confirmation of CRISPR/Cas9-mediated deletions

Confirmation of *STA1* deletion (-1370 to +2421 relative to *STA1* start codon):

TUM PI BA 109_S1 / TUM 71_S1 / WY3711_S1

CGTAGCCTTG TCAACTTAGA CTCAGTTCCA CGGCGTGCAG GACGGGGTAT TATGAATAAA GGATCCACGG GTAAGATTTG ACAAAAAAAA ATAAAAGAAA AGCGAGAAGT ATACACAAGT GTATTTCCTA GATATTTACA TCAAATATAT ATATATATAC

GACGGGGTAT TATGAATAAA GGATCCACGG GTAAGATTTG ACAAAAAAAA ATAAAAGAAA AGCGAGAAGT ATACACAAGT

Repair oligo (repair_oligo_orf_deletion)

Legend:
Pre-gap sequence
Post-gap sequence
Repair oligo

Confirmation of deletion in *STA1* promoter (-1370 to -209 relative to *STA1* start codon):

WY3711_D1

CGTAGCCTTG TCAACTTAGA CTCAGTTCCA CGGCGTGCAG GACGGGGTAT TATGAATAAA GGATCCACGG GTAAGATTTG CTGCGCTCTC TTCTAGTTCA AGAACGGATA ACTCATAGAC TTACCTGTAC AAGTTGTTGA AGGGTTCTCA ATTGATAAAA

GACGGGGTAT TATGAATAAA GGATCCACGG GTAAGATTTG CTGCGCTCTC TTCTAGTTCA AGAACGGATA ACTCATAGAC

Repair oligo (repair_oligo_promoter_deletion)

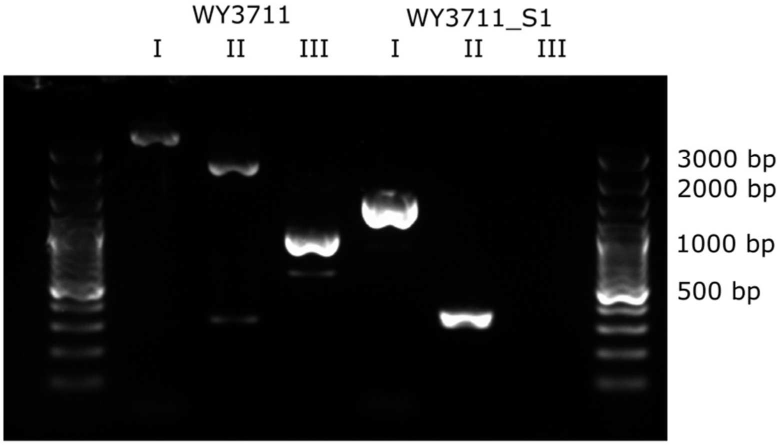

Figure S1 – Confirmation of CRISPR/Cas9-mediated deletions by Sanger sequencing and PCR. Primers used to confirm the deletion of *STA1* (-1370 to +2421 relative to start codon) by PCR: **I**: STA1_Full_Fw / STA1_Full_Rv, **II**: STA1_1055_F / STA1_5201_R, **III**: SD-5A / SD-6B

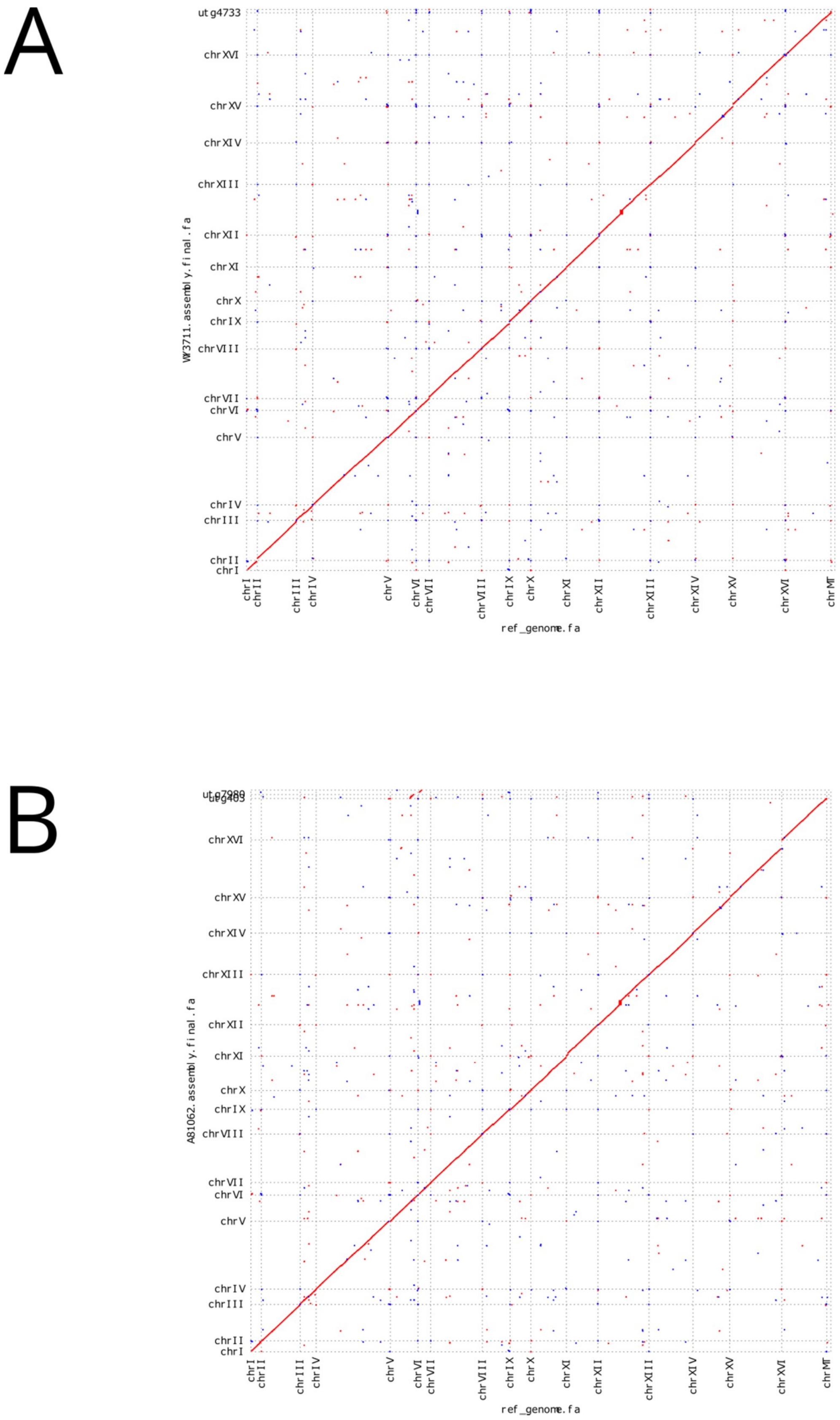

Figure S2 – Comparison of (**A**) *S. cerevisiae* WY3711 and (**B**) *S. cerevisiae* A81062 *de novo* assemblies with *S. cerevisiae* S288C reference genome.

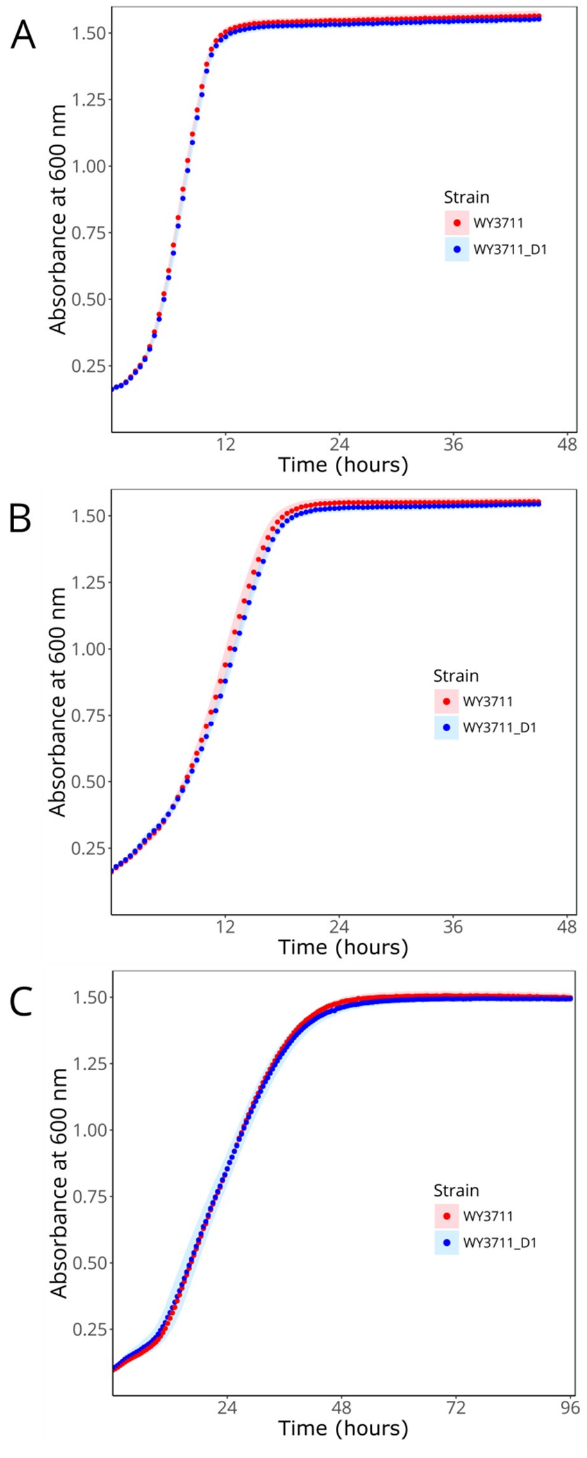

Figure S3 – The growth (absorbance at 600 nm) of *S. cerevisiae* WY3711 (red) and WY3711_D1 (blue) in **(A)** YP-Glucose (1%), (**B**) YP-Maltose (1%), and (**C**) YNB-Maltotriose (1%). Cultivations were performed in microplate format at 25 °C. Points and shaded areas represent the mean and standard deviation of 8 biological replicates per strain, respectively. No significant difference was observed between the two strains in any media (two-tailed Student's *t*-test, $p > 0.05$).

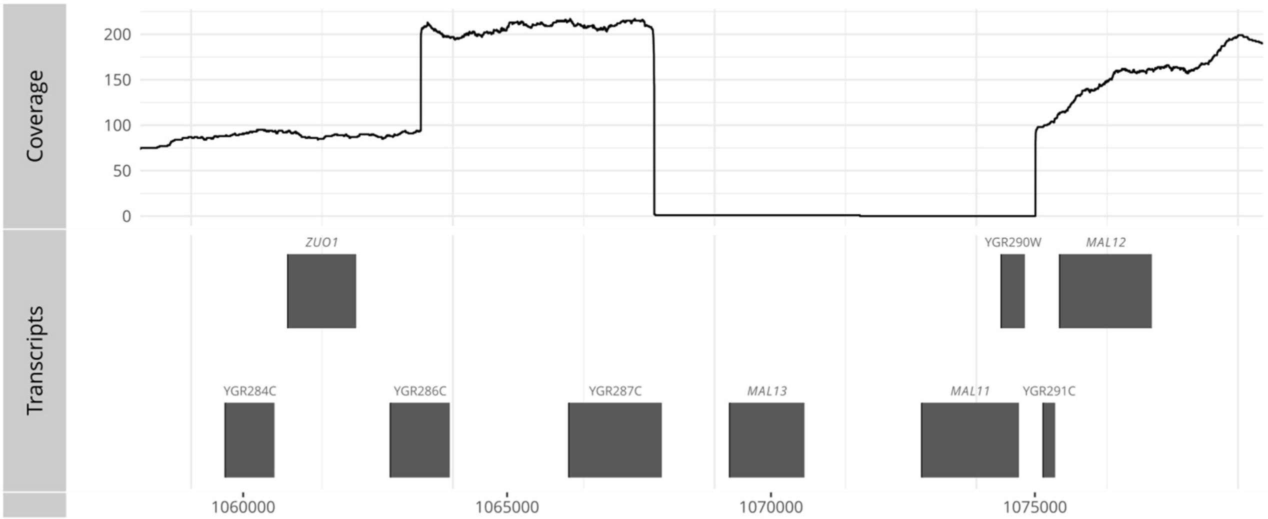

Figure S4 – The sequencing coverage of Nanopore reads from *S. cerevisiae* WY3711 aligned to *S. cerevisiae* S288C around the *MAL1* locus (chromosome VII: 1060000-1080000).

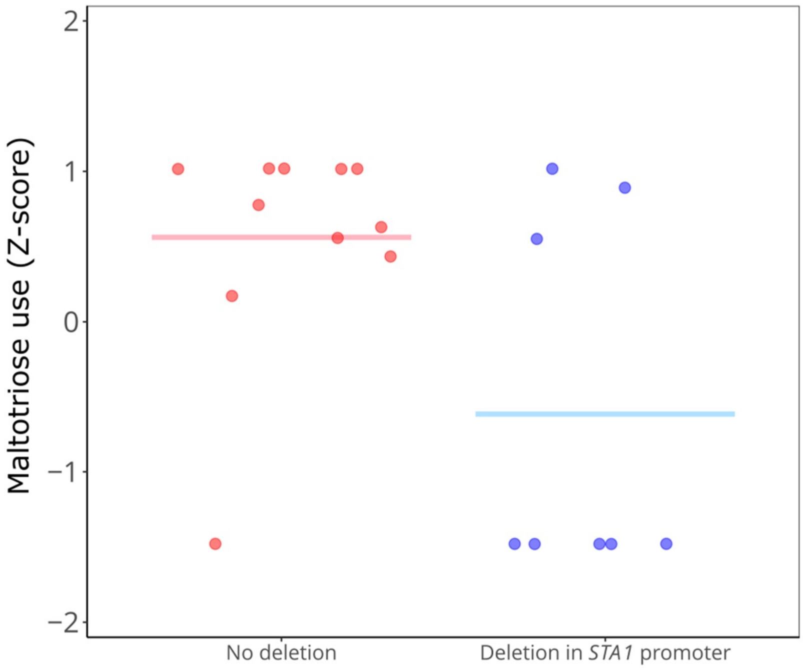

Figure S5 – The ability to use maltotriose among the *STA1+* strains studied by Gallone et al. (2016). Strains are grouped depending on whether they have an 1162 bp deletion in the *STA1* promoter. Z-scores were obtained from Supplementary Table S5 in Gallone et al. (2016). The group average is depicted as a straight line. The two groups differed significantly (Mann-Whitney U test, $p = 0.045$).

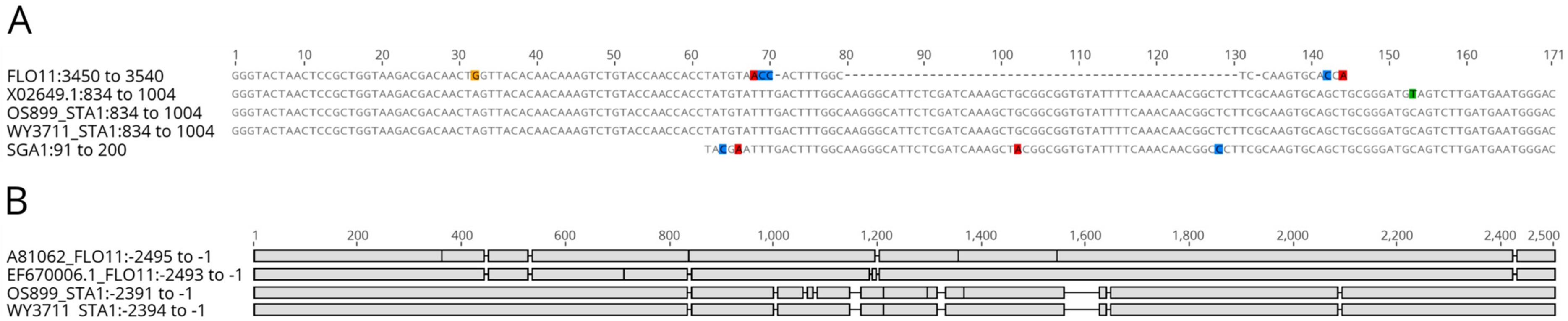

Figure S6 – Multiple sequence alignment of (**A**) sequences around the *FLO11*/*SGA1* junction in *STA1* (GenBank X02649.1) from *S. cerevisiae* WY3711 (‘Beer 2’/’Mosaic Beer’) and *S. cerevisiae* OS899 (‘French Guiana human’), and (**B**) sequences upstream of *STA1* from *S. cerevisiae* WY3711 (‘Beer 2’/’Mosaic Beer’) and *S. cerevisiae* OS899 (‘French Guiana human’) and *FLO11* (GenBank EF670006.1) from *S. cerevisiae* A81062 (‘Beer 2’/’Mosaic Beer’).

Supplementary Table S1 – The assembly statistics for *S. cerevisiae* WY3711 and A81062

	**WY3711**	**A81062**
Total sequence count	17	18
Total sequence length	12337335	12525172
Min sequence length	75369	83792
Max sequence length	1479817	1507828
Mean sequence length	725726	695843
Median sequence length	749327	735604
N50	910883	918893
L50	6	6
N90	448377	424662
L90	13	13
A%	30.79	30.85
T%	30.73	30.77
G%	19.23	19.16
C%	19.26	19.22
AT%	61.52	61.62
GC%	38.48	38.38
N%	0.00	0.00

Supplementary Table S2 – Accession numbers or links to genome assemblies, short sequencing reads, and long sequencing reads of three *STA1+* strains.

Strain name	Short-read genome assembly	Short sequencing reads	Long sequencing reads
A81062	ASM193724v1 (https://www.ncbi.nlm.nih.gov/assembly/GCA_001937245.1/)	SRX1423875	SRX1423868
WLP570	https://www.yeastgenome.org/1011-yeast-genomes (strain CFF)	ERX1380425	ERX1609847
OS899	https://www.yeastgenome.org/1011-yeast-genomes (strain BCN)	ERR1308824	ERX1609841

Supplementary Table S3 – The sequences used to query for the presence of *STA1* and the 1162 bp deletion in the *STA1* promoter.

**Name**	**Sequence**
STA1_BLAST	TTCCAACTGCACTAGTTCCTAGAGGATCCTCCTCTAGCAAC<br>ATCACTTCCTCCGGTCCATCTTCAACTCCATTCAGCTC
STA1_deletion_BLAST	TGCATTTCCCAAAATTCATTCGTAGCCTTGTCAACTTAGAC<br>TCAGTTCCACGGCGTGCAGGACGGGGTATTATGAATAAAG<br>GATCCACGGGTAAGATTTGCTGCGCTCTCTTCTAGTTCAAG<br>AACGGATAACTCATAGACTTACCTGTACAAGTTGTTGAAG<br>GGTTCTCAATTGATAAAAAAGGATCTTTTGCTTCCTAA

Supplementary Table S4 – Estimated copy numbers of the open reading frame and deleted region in the promoter of *STA1* in the 'Beer 2'/'Mosaic Beer' *S. cerevisiae* strains. Copy numbers are estimated by normalizing the coverage in two unique regions in the *STA1* promoter and ORF to that of the whole genome. A normalized coverage of **0 indicates a homozygous deletion in the *STA1* promoter**, **0.5 indicates a hemizygous *STA1* allele or hemi-/heterozygous deletion in the *STA1* promoter**, 1 indicates a homozygous *STA1* allele or full promoter, and **above 1.5 indicates a duplication of *STA1***.

Strain	NCBI-SRA accession number	Median coverage			Normalized coverage	
		Whole genome	*STA1* promoter (-923 to -823)	*STA1* ORF (+65 to +143)	*STA1* promoter (-923 to -823)	*STA1* ORF (+65 to +143)
AAQ	ERR1309237	160	189	173	1.2	1.1
AAR	ERR1308657	210	220	195	1.0	0.9
AEA	ERR1309406	203	475	547	2.3	2.7
AEQ	ERR1309512	214	217	241	1.0	1.1
AFA	ERR1309420	193	239	233	1.2	1.2
AFB	ERR1308680	213	248	221	1.2	1.0
AFP	ERR1309364	237	278	232	1.2	1.0
AQG	ERR1308609	223	266	284	1.2	1.3
AQH	ERR1309146	217	230	227	1.1	1.0
Beer002	SRR5678585	104	152	120	1.5	1.2
Beer004	SRR5678609	118	0	66	0.0	0.6
Beer011	SRR5678570	90	0	45	0.0	0.5
Beer013	SRR5678568	120	57	253	0.5	2.1
Beer021	SRR5678680	77	73	85	1.0	1.1
Beer032	SRR5678684	78	48	40	0.6	0.5
Beer034	SRR5678686	86	0	74	0.0	0.9
Beer039	SRR5678681	121	0	58	0.0	0.5
Beer040	SRR5678682	77	0	31	0.0	0.4
Beer059	SRR5688171	70	24	111	0.3	1.6
Beer062	SRR5688177	113	145	156	1.3	1.4
Beer080	SRR5688213	97	89	71	0.9	0.7
Beer083	SRR5688219	51	0	47	0.0	0.9
Beer084	SRR5688221	131	3	210	0.0	1.6
Beer085	SRR5688223	231	2	208	0.0	0.9
Beer086	SRR5688225	125	116	168	0.9	1.3
Beer091	SRR5688235	170	90	133	0.5	0.8
Beer092	SRR5688237	123	172	188	1.4	1.5
BRM	ERR1309517	201	391	406	1.9	2.0
CFF	ERR1309102	241	0	86	0.0	0.4
Wine019	SRR5688275	82	110	111	1.3	1.4

Supplementary Table S5 – BLAST results for *STA1* (Genbank X02649.1). The full-length hits have been highlighted with a light grey background.

Strain	Contig	% Identity	Alignment length	Mis-matches	Gap opens	Query start	Query end	Subject start	Subject end	e-value	bit score
**Short-read genome assemblies**											
A81062	Scerevisiae_A62_chromosome_IX	98.888	1439	15	1	900	2337	164632	166070	0	2567
	Scerevisiae_A62_chromosome_IX	97.738	619	14	0	282	900	372406	371788	0	1066
	Scerevisiae_A62_chromosome_IX	97.362	417	11	0	4	420	372816	372400	0	710
WLP570	CFF_4-19034	99.027	1439	13	1	900	2337	2004	566	0	2579
	CFF_4-19035	98.703	848	11	0	2	849	1659	2506	0	1506
	CFF_4-19163	97.9	619	13	0	282	900	20571	19953	0	1072
OS899	BCN_8-7617	99.305	1439	9	1	900	2337	5427	3989	0	2601
	BCN_8-7599	100	686	0	0	179	864	1	686	0	1267
**Long-read genome assemblies**											
**Strain**	**Contig**	**% Identity**	**Alignment length**	**Mis-matches**	**Gap opens**	**Query start**	**Query end**	**Subject start**	**Subject end**	**e-value**	**bit score**
A81062	A81062_chrIX	99.358	2337	14	1	2	2337	423717	421381	0	4231
	A81062_chrIX	98.888	1439	15	1	900	2337	166095	167533	0	2567
	A81062_chrIX	97.258	620	16	1	282	900	392537	391918	0	1050
	A81062_chrIX	96.172	209	8	0	212	420	392739	392531	1.92E-93	342
WLP570	CFF_chrX	97.523	2342	21	27	2	2333	731041	728727	0	3969
	CFF_chrIX	98.75	1440	16	2	900	2337	166688	168127	0	2558
	CFF_chrIX	97.558	778	19	0	123	900	380269	379492	0	1332
OS899	BCN_chrII	99.872	2338	2	1	1	2337	16056	18393	0	4300
	BCN_chrIX	98.749	1439	17	1	900	2337	166148	167586	0	2556
	BCN_chrXIII	99.743	778	2	0	123	900	673907	673130	0	1426
	BCN_chrX	99.357	778	2	3	123	900	186401	187175	0	1406
	BCN_chrX	74.57	582	116	19	323	900	186919	187472	1.91E-58	226
	BCN_chrXIII	74.394	578	116	18	323	896	673386	672837	3.19E-56	219

Supplementary Table S6 – BLAST results for 'STA1_BLAST' (Supplementary Table S2) in the 1169 *S. cerevisiae* genome assemblies from Gallone et al. (2016) and Peter et al. (2018).

Strain	Contig	% Identity	Alignment length	Mis-matches	Gap opens	Query start	Query end	Subject start	Subject end	e-value	bit score
AAQ	AAQ_3-7332	100	79	0	0	1	79	695	773	5.66E-34	147
AAR	AAR_3-7571	100	79	0	0	1	79	767	689	5.66E-34	147
AEA	AEA_8-6787	100	79	0	0	1	79	767	689	5.66E-34	147
AEQ	AEQ_3-11697	100	79	0	0	1	79	695	773	5.66E-34	147
AFA	AFA_4-7853	100	79	0	0	1	79	3661	3583	5.66E-34	147
AFB	AFB_4-8105	100	79	0	0	1	79	580	502	5.66E-34	147
AFP	AFP_1-7540	100	79	0	0	1	79	3659	3581	5.66E-34	147
ALI	ALI_5-7132	100	79	0	0	1	79	88	10	5.66E-34	147
AQG	AQG_4-15855	100	79	0	0	1	79	767	689	5.66E-34	147
AQH	AQH_2-39734	100	79	0	0	1	79	958	1036	5.66E-34	147
BCB	BCB_3-7948	100	79	0	0	1	79	88	10	5.66E-34	147
BCC	BCC_3-6681	100	79	0	0	1	79	88	10	5.66E-34	147
BCE	BCE_8-6067	100	79	0	0	1	79	32	110	5.66E-34	147
BCF	BCF_8-6617	100	79	0	0	1	79	88	10	5.66E-34	147
BCI	BCI_8-6529	100	79	0	0	1	79	32	110	5.66E-34	147
BCK	BCK_8-7150	100	79	0	0	1	79	32	110	5.66E-34	147
BCL	BCL_5-6462	100	79	0	0	1	79	32	110	5.66E-34	147
BCN	BCN_8-4221	100	79	0	0	1	79	32	110	5.66E-34	147
BCP	BCP_8-5410	100	79	0	0	1	79	88	10	5.66E-34	147
BDP	BDP_5-6002	100	79	0	0	1	79	88	10	5.66E-34	147
BDQ	BDQ_6-7824	100	79	0	0	1	79	32	110	5.66E-34	147
BDR	BDR_6-6225	100	79	0	0	1	79	32	110	5.66E-34	147
BDS	BDS_6-8316	100	79	0	0	1	79	41	119	5.66E-34	147
BDT	BDT_6-11671	100	79	0	0	1	79	767	689	5.66E-34	147
BEB	BEB_6_C37T3ACXX.IND41b-7767	100	79	0	0	1	79	88	10	5.66E-34	147

BED	BED_6-6933	100	79	0	0	1	79	88	10	5.66E-34	147
beer002	MCAA01000038.1	100	79	0	0	1	79	3980	4058	5.66E-34	147
beer004	CM007127.1	100	79	0	0	1	79	398847	398769	5.66E-34	147
beer011	CM007015.1	100	79	0	0	1	79	382411	382333	5.66E-34	147
beer013	CM006967.1	100	79	0	0	1	79	396002	395924	5.66E-34	147
beer021	CM006855.1	100	79	0	0	1	79	397405	397327	5.66E-34	147
beer032	CM006663.1	100	79	0	0	1	79	395834	395756	5.66E-34	147
beer034	CM006647.1	100	79	0	0	1	79	399862	399784	5.66E-34	147
beer039	CM006567.1	100	79	0	0	1	79	392082	392004	5.66E-34	147
beer040	CM006551.1	100	79	0	0	1	79	387784	387706	5.66E-34	147
beer059	CM006247.1	100	79	0	0	1	79	186841	186919	5.66E-34	147
beer062	CM006199.1	100	79	0	0	1	79	392434	392356	5.66E-34	147
beer080	CM005911.1	100	79	0	0	1	79	408118	408040	5.66E-34	147
beer083	CM005863.1	100	79	0	0	1	79	404330	404252	5.66E-34	147
beer084	MBWW01000093.1	100	79	0	0	1	79	1143	1065	5.66E-34	147
beer085	CM005831.1	100	79	0	0	1	79	396106	396028	5.66E-34	147
beer086	CM005815.1	100	79	0	0	1	79	400774	400696	5.66E-34	147
beer091	CM005735.1	100	79	0	0	1	79	393037	392959	5.66E-34	147
beer092	CM005719.1	100	79	0	0	1	79	402761	402683	5.66E-34	147
BMV	BMV_2-6044	100	79	0	0	1	79	88	10	5.66E-34	147
BNA	BNA_4-6466	100	79	0	0	1	79	32	110	5.66E-34	147
BNC	BNC_4-9323	100	79	0	0	1	79	88	10	5.66E-34	147
BRM	BRM_4-7163	100	79	0	0	1	79	6097	6019	5.66E-34	147
CFF	CFF_4-19035	100	79	0	0	1	79	1755	1833	5.66E-34	147
CRE	CRE_2-37810	100	79	0	0	1	79	752	674	5.66E-34	147
spirits007	CM005159.1	100	79	0	0	1	79	392416	392338	5.66E-34	147
spirits008	CM005143.1	100	79	0	0	1	79	408577	408499	5.66E-34	147
wine019	CM004807.1	100	79	0	0	1	79	180241	180319	5.66E-34	147
YAB	YAB-7810	100	79	0	0	1	79	700	778	5.66E-34	147

Supplementary Table S7 – BLAST results for *MAL11*, *MAL31* and *MTT1* (GenBank LT594281) in *S. cerevisiae* WY3711. Hits from different queries with the same letter in final column (*) are the same subject sequence.

Query	Contig	% Identity	Alignment length	Mis-matches	Gap opens	Query start	Query end	Subject start	Subject end	e-value	bit score	*
MAL11	0 hits found											
MAL31	chrVII	97.243	1850	37	14	1	1845	1094456	1092616	0	3121	a
MAL31	chrII	91.905	1853	139	8	1	1845	856240	854391	0	2580	b
MAL31	chrIII	91.707	1857	135	16	1	1845	322468	320619	0	2558	c
MAL31	chrXI	91.604	1858	137	16	1	1845	655300	653449	0	2549	d
MAL31	chrII	90.081	1855	170	11	1	1845	840081	838231	0	2394	e
MTT1 (LT594281)	chrII	97.896	1854	30	8	1	1848	840081	838231	0	3199	e
MTT1 (LT594281)	chrIII	95.356	1852	80	6	1	1848	322468	320619	0	2939	c
MTT1 (LT594281)	chrXI	95.038	1854	84	8	1	1848	655300	653449	0	2907	d
MTT1 (LT594281)	chrII	94.941	1858	76	15	1	1848	856240	854391	0	2894	b
MTT1 (LT594281)	chrVII	89.32	1854	179	16	1	1848	1094456	1092616	0	2309	a
